# Continuous glucose monitoring in neonates: a review

**DOI:** 10.1186/s40748-017-0055-z

**Published:** 2017-10-17

**Authors:** Christopher J.D. McKinlay, J. Geoffrey Chase, Jennifer Dickson, Deborah L. Harris, Jane M. Alsweiler, Jane E. Harding

**Affiliations:** 1 Liggins Institute, University of Auckland, Private Bag 92019, Victoria St West, Auckland, 1142 New Zealand; 2 Department of Paediatrics: Child and Youth Health, University of Auckland, Auckland, New Zealand; 3 Mechanical Engineering, University of Canterbury, Christchurch, New Zealand; 4 Neonatal Intensive Care Unit, Waikato District Health Board, Hamilton, New Zealand

**Keywords:** Neonatal hypoglycaemia, Neonatal hyperglycaemia, Interstitial glucose, Continuous glucose monitoring, Hyperinsulinaemia

## Abstract

Continuous glucose monitoring (CGM) is well established in the management of diabetes mellitus, but its role in neonatal glycaemic control is less clear. CGM has provided important insights about neonatal glucose metabolism, and there is increasing interest in its clinical use, particularly in preterm neonates and in those in whom glucose control is difficult. Neonatal glucose instability, including hypoglycaemia and hyperglycaemia, has been associated with poorer neurodevelopment, and CGM offers the possibility of adjusting treatment in real time to account for individual metabolic requirements while reducing the number of blood tests required, potentially improving long-term outcomes. However, current devices are optimised for use at relatively high glucose concentrations, and several technical issues need to be resolved before real-time CGM can be recommended for routine neonatal care. These include: 1) limited point accuracy, especially at low or rapidly changing glucose concentrations; 2) calibration methods that are designed for higher glucose concentrations of children and adults, and not for neonates; 3) sensor drift, which is under-recognised; and 4) the need for dynamic and integrated metrics that can be related to long-term neurodevelopmental outcomes. CGM remains an important tool for retrospective investigation of neonatal glycaemia and the effect of different treatments on glucose metabolism. However, at present CGM should be limited to research studies, and should only be introduced into routine clinical care once benefit is demonstrated in randomised trials.

## Background

Continuous glucose monitoring (CGM) is well established in the management of diabetes mellitus, but its role in neonatal care is less clear. CGM has provided important insights about neonatal glucose metabolism [1, 2], and there is increasing interest in its clinical use, particularly in preterm neonates and in those in whom glucose control is difficult. Neonatal glucose instability, including hypoglycaemia and hyperglycaemia, has been associated with poorer neurodevelopment [3], and serial blood glucose monitoring by heel lancing is invasive, with potential adverse effects on neurodevelopment [4]. CGM offers the possibility of adjusting treatment in real time to account for individual metabolic requirements while reducing the number of blood glucose measurements required, potentially improving long-term outcomes [5]. However, several technical issues need to be resolved before CGM can be recommended for routine neonatal care, including accuracy, calibration, sensor drift and plasma-interstitial time delay.

The clinical interpretation of CGM is also challenging. Neonatal studies using CGM have revealed that variability in glucose concentrations is common both during neonatal transition [1, 3] and in enterally fed preterm infants [5–9]. However, the clinical significance of these findings is uncertain, and in the absence of well-established guidelines there is a risk that CGM could lead to unnecessary or even harmful intervention [3]. Further, while CGM provides more information than intermittent blood testing, it is also less accurate. CGM parameters, therefore, need to be conceptualised as dynamic and integrated rather than as static thresholds, and there is little information about how such parameters should be interpreted.

It is important that clinicians understand the limitations and implications of this rapidly evolving technology before it is adopted into clinical practice. This paper will review: 1) CGM technologies for neonatal care; 2) insights from CGM about neonatal glucose metabolism; and 3) the current evidence for the clinical application of CGM in neonatal intensive care.

## CGM technology for neonatal care

### Types of CGM

CGM devices measure the glucose concentration of the interstitial fluid, either in subcutaneous tissue or in transdermal fluid (Table 1). Subcutaneous biosensors are of two types: microdialysis fibres and amperometric needle electrodes. Microdialysis involves insertion into the subcutaneous tissue of a thin hollow fibre that is composed of a semipermeable membrane through which an isotonic fluid containing no glucose is infused [10]. Glucose from the interstitium diffuses into the fluid stream and is measured by an external enzymatic probe. These devices have had only limited use in neonates in research settings [11, 12], and there are currently no commercial systems available.

**Table 1:** Methodologies for continuous glucose monitoring

Fluid location	Biosensor	Advantages	Disadvantages	Commercial devices currently used in neonates
Subcutaneous	Microdialysis fibre with external amperometric probe.	Most accurate.	Subcutaneous inflammation.	Not available
		Sensing element is outside the skin and so is not susceptible to biofouling.		
			Expensive.	
			Long lag time.	
			Discomfort.	
			Requires calibration.	
	Amperometric needle electrode.	Easier insertion.	Less accurate.	Medtronic MiniMed.
			Sensor degradation due to biofouling.	
				DexCom.
			Poor detection with oedema.	
			Discomfort. Most require calibration.	
Transdermal	Glucose binding protein.	No skin penetration.	Accuracy unknown.	Not yet available.
		Potentially suitable in neonates due to their high trans-epidermal water loss.		

Subcutaneous needle CGM systems consist of a fine needle sensor connected to a non-implantable transmitter that powers the sensor and sends raw data to a monitor, either by cable or Bluetooth. Some systems display the resulting output in real-time on the monitor or another linked device; others store the data for later downloading. The challenge for sensor manufacturers is to combine all the components of an enzymatic ampometric system into a single needle [13]. Ampometric sensors measure current flowing from an oxidation (electron producing) reaction at a working electrode to a reduction (electron consuming) reaction at a counter electrode. The working electrode is coated with glucose oxidase which catalyses the oxidation of glucose when a voltage is applied, resulting in transfer of electrons to a chemical mediator, usually hydrogen peroxide. A reference electrode is used to ensure a stable voltage is applied to the working electrode, but reference and counter electrodes are often combined. In addition, subcutaneous sensors require a barrier membrane to limit glucose access to the sensor because of the deficiency of oxygen in the subcutaneous environment relative to glucose supply. Each manufacturer has their own proprietary method for combining these elements within the needle sensor.

**Fig. 1:**
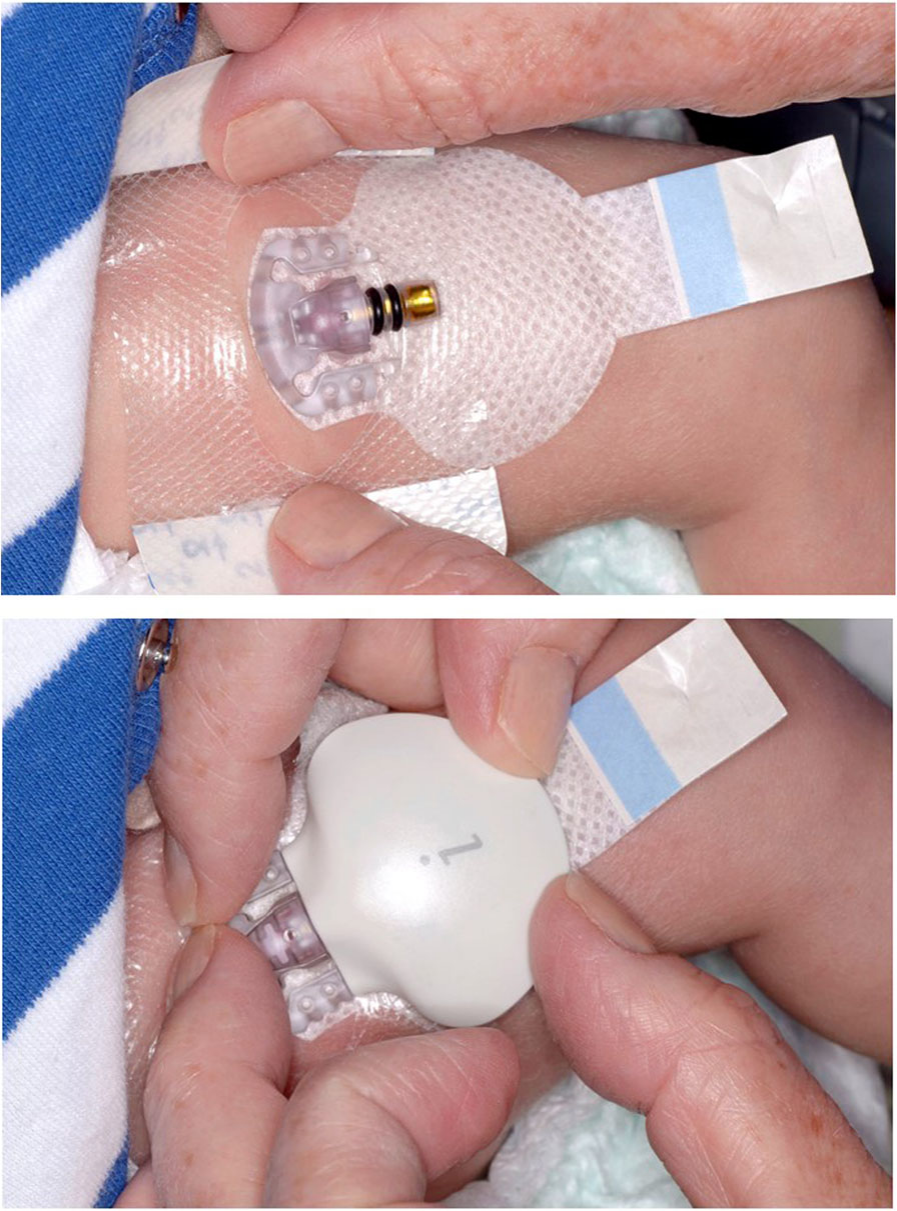
Insertion of a continuous glucose sensor and attachment of transmitter in the lateral thigh of a newborn infant

Two main CGM brands have been used in neonates, Medtronic Minimed (Northridge, CA, United States) and DexCom (San Diego, CA, United States), both of which manufacture retrospective and real-time devices. However, it should be noted that none of these devices have been approved for clinical use in neonates. The needle electrodes and transmitters have generally been placed on the lateral thigh in neonates (Fig. 1), and have been used for up to 7 days [1, 14]. Some, but not all babies appear to experience brief pain on insertion of the needle electrode, but sensors are subsequently well tolerated in most neonates, and complications are rare [1, 15].

Raw signal data from the electrode is generated approximately every 10 s, and is averaged and processed to give a glucose reading every 5 min, thus providing near-continuous output. Notably, these devices do not display data when glucose concentrations fall below 2.2 mmol/L (40 mg/dl), although retrospective analysis and calibration of the raw signal may still be possible [16]. Subcutaneous devices may also fail to give readings in the presence of significant skin oedema or dehydration, and with use of vasopressors.

Conversion of the raw signal to a glucose result requires regular input of calibration measures, i.e., current blood or plasma glucose concentrations, and thus the resulting output will reflect the calibration samples used. It is, therefore, important to understand the effect of sample type, collection method, analytical delay and assay technique on calibration values. For example, glucose concentration in whole-blood samples is usually reported to be lower than in plasma. However, immediate testing of whole arterial or capillary blood with a ward-based blood gas analysers can yield very similar glucose concentrations to laboratory analysis of plasma derived from the same samples, with a mean processing delay of 30 min [17].

Transdermal sensors are still in development, but in the future, may be useful in neonates because of their thin skin layer [18]. They can be divided into those that rely on passive diffusion of glucose into the transdermal fluid and those that use reverse iontophoresis to induce flow of molecules through the skin by applying a small electrical surface current. Because the concentration of glucose in the transdermal fluid is very low, signal transduction relies on specialised glucose binding proteins that undergo conformational change in the presence of glucose.

### Accuracy and types of error

**Fig. 2:**
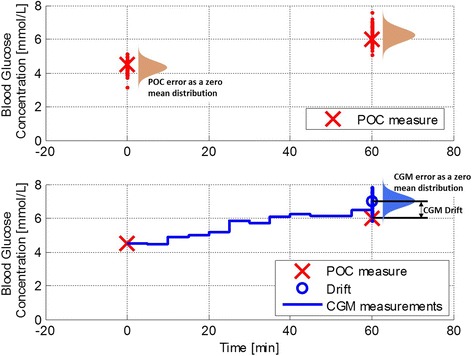
Comparison of the types of measurement error for point-of-care (POC) and continuous glucose monitors (CGM), where CGMs can be prone to drift as well as a zero-centred noise

Like all glucose sensors, CGM accuracy is affected by random error or noise, which may vary with glucose concentration [19]. Commonly used point-of-care glucometers typically have a zero-mean error (deviation from the true value) of 10% to 30% [20, 21]. However, CGM error also contains a drift component (Fig. 2) [22]. CGM measures rely on a continuous shifting internal algorithm to generate a glucose concentration from the raw sensor signal, based on regular calibration against ‘true’ measurements (blood sample) which are entered into the device by the clinician or patient. In addition to random error, the reported glucose concentration can ‘drift’ from the true measure between calibration measurements and this may significantly impact on accuracy, particularly if there is a sudden change in glycaemic status [16]. Most devices require calibration input at least every 12 h, with more frequent calibration recommended for increased accuracy.

While random, zero-mean errors can be large in CGMs due their interstitial location and sensing method, drift is unseen and thus a major issue in monitoring and control. For example, drift may result in an apparent constant CGM glucose reading when blood glucose concentration is actually falling. Perhaps worse, drift is generally not quantified as it is not required for regulatory approval, even though it has been shown to be problematic in a number of CGM devices [16, 23]. Standard assessment statistics for sensors, such as mean absolute relative difference (MARD) [24], indicate overall error but do not delineate its various components.

A typical CGM device has a MARD of 7% to 12% [23, 25], which, in adults, can vary based on the location of the sensor and acuity of the patient [26]. Assessment of drift requires intermediate independent glucose measurements, but this undermines one of the key benefits of CGM, namely, reduced blood sampling. Further, it is likely that the level of drift changes in different clinical situations, and so it is difficult to be certain about error limits. Nevertheless, expected ranges have been modelled for several sensors and devices in adult cohorts [23, 27].

Most CGM devices are designed for type 1 or type 2 diabetes and use a multiple point weighted calibration, i.e., the algorithm takes into account a weighted average of the last several ‘true’ calibrating glucose concentrations entered. Multiple point calibration aims to increase accuracy over the range of calibration points recorded because it is assumed that less accurate point-of-care glucometers will be used for calibration [28]. However, when the blood glucose concentration deviates from the previous range of calibration values, calibration may be less accurate and errors may increase, particularly at low glucose concentrations [16].

Sensor drift results from altered access to interstitial fluid or a change in probe surface due to biofilm build up or corrosion, so that different currents are generated by the same blood glucose concentration. Multiple point calibration exacerbates this problem as each measurement can influence calibration for longer. For example, if a device is calibrated every 8 h using a three-point calibration method, each measure will influence calibration for up to 24 h.

**Fig. 3:**
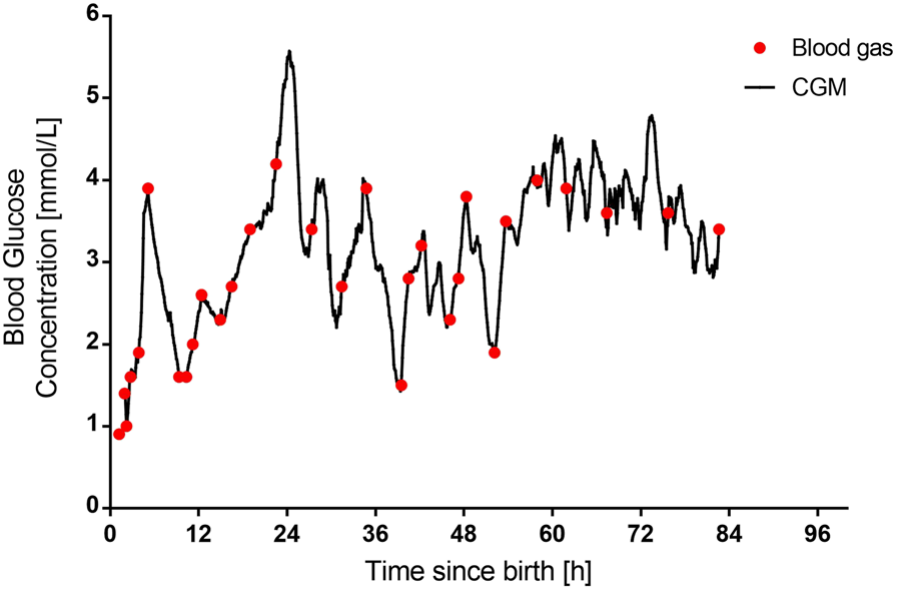
Example of neonatal subcutaneous continuous glucose monitoring (CGM) with retrospective point-to-point calibration (Data from McKinlay et al. [3])

In point-to-point calibration, the algorithm interpolates readings only between one calibration glucose measure and the next. This avoids the problem of multiple point calibration exacerbating the error due to sensor drift, and is suitable for neonatal intensive care where highly accurate glucose measurements are readily available from a blood gas machine or laboratory analyser (Fig. 3) [16], However, point-to-point calibration is not employed in currently available real-time CGM devices for clinical care. Rather, current devices use proprietary algorithms based on multiple calibration measures, and the calibration method can only be changed by the manufacturer. Fortunately, some retrospective devices, such as that produced by Medtronic MiniMed, output the actual sensor current, which is very useful in research because it allows the researcher to apply post hoc point-to-point recalibration to reduce calibration error and ameliorate drift [16].

An additional challenge in achieving accurate CGM measurements is that calibration must account for the diffusion of glucose from blood to interstitial fluid. This imposes not only a variable time delay, but also a low-pass filter effect, altering the glucose concentration dynamics between blood and interstitial fluid [29]. Further, time delay tends to increase as blood glucose concentration falls [30, 31], so that there is usually increasing positive error (CGM reading higher than true blood glucose concentration) at onset of hypoglycaemia [32]. This could lead to delayed intervention if CGM were to be used clinically to monitor for hypoglycaemia. Little is known of blood-interstitial glucose dynamics in neonates, although lag times of up to 1 h have been reported [33].

### Analysis and display of CGM data

Given the measurement errors inherent in real-time CGM, it cannot be relied upon for point accuracy, particularly when looking for outlying events, such as hypoglycaemia. Further, traditional glycaemic metrics based on exceeding a specified threshold, such as hypoglycaemic episodes, have limited utility because of the potentially large zero-mean, random errors. For example, in very low birthweight infants, real-time CGM, had a positive predictive value for hypoglycaemia (blood glucose <2.6 mmol/L [<47 mg/dl]) of only 40%, and wide limits of agreement (~ ± 1.5 mmol/L [± 27 mg/dl]) [14]. In another study that measured blood glucose concentration only by point-of-care glucometer, the limits of agreement for CGM were even wider (~ ± 2.0 mmol/L [± 36 mg/dl]) [34]. Nevertheless, CGM, with its high density of data, contains a wealth of information about glycaemic status that may be of clinical importance.

An alternative approach is to focus on clusters of data points in moving windows. Although this reduces the number of independent ‘measures’, it minimises the impact of random errors, while still providing more frequent information than intermittent blood sampling. Direct filtering is also possible in post hoc analysis [16], but this is currently not available in real time. Guidelines for neonatal CGM analysis are yet to be established, but four main types of metrics should be considered:

i. Time within a desirable band. Studies in adult intensive care patients suggest that the cumulative quality of blood glucose control is physiologically more important than the number of extreme excursions. For example, patients who spend more time in the central blood glucose range appear to have less organ injury and better survival [35–37], although the extent to which these associations are causal is a focus of ongoing debate.
ii. Integral (area) under the glycaemic curve. This can be useful for creating alarm or guardrail systems to warn of impending clinically significant events. For example, a diminishing integral has been shown to be a better marker of impending hypoglycaemia than a rolling point average [38].
iii. Rising or falling trend. This is the rate of change over time, which can also be used to warn that a clinical threshold is about to be exceeded. It should be noted that the accuracy of trend information is different to point accuracy, and must be evaluated and monitored separately. One such tool, the Trend Compass, appears to be suitable for this purpose [39].
iv. Point-to-point change. In general, changes between each individual CGM measure should be relatively small. The distribution of these changes can be used to determine unlikely or outlying changes in sensor output [40, 41]. A large number or cluster of such extreme point-to-point changes can indicate sensor failure or a marked change in subcutaneous tissue condition, such as with oedema. Alternatively, they could be used to detect periodic events that induce larger glucose changes, such as feeding [41].

## Insights from CGM about neonatal glucose metabolism

### Healthy term infants

It is commonly reported that neonatal glucose concentrations decline after birth, reaching a physiological nadir at approximately 2 h [42]. However, several serial [43] and cross-sectional [44] studies in healthy, breastfed term infants have failed to demonstrate any nadir in true (laboratory) blood glucose concentrations. On the contrary, these studies suggest, albeit based on intermittent sampling, that mean blood glucose concentration remains stable at approximately 3.0 mmol/L (54 mg/dl) throughout the first 24 to 48 h, thereafter gradually rising to a mean of approximately 4.0 mmol/L (72 mg/dl) [45].

CGM offers the possibility of studying early trends in neonatal glycaemia in greater detail, including effects of feeding. Several studies have shown that this is feasible from shortly after birth, although delays between birth and sensor insertion, and the period of sensor “wetting” before the output signal stabilises, means that it is difficult to get reliable measurements in the first 2 h [1, 46]. A study is currently under way in healthy term infants that is using CGM with retrospective point-to-point recalibration based on true blood glucose concentrations, and will provide important new insights about glucose dynamics during the neonatal transition (http://hdl.handle.net/2292/32066). This is important because it has been argued that early transitional hypoglycaemia is a normal physiological phenomenon, and so does not require treatment. However, in one population study, even a single episode of neonatal hypoglycaemia was associated with poorer performance on later learning, raising the question of whether all newborns should be screened for hypoglycaemia rather than just those with risk factors for impaired metabolic adaptation [47].

### At-risk term and near-term infants

A large, prospective cohort study of term and late preterm infants born at risk of neonatal hypoglycaemia (the CHYLD Study) included retrospective CGM data for ≥48 h in 75% of the cohort [3]. Analysis was based on point-to-point recalibration using all available blood glucose measures (all by blood gas analyser), thus maximising accuracy at low glucose concentrations [16]. This study showed that despite regular blood glucose testing and a clinical management protocol aimed at maintaining blood glucose ≥2.6 mmol/L [≥47 mg/dl], many infants experienced prolonged periods of low interstitial glucose (<2.6 mmol/L [<47 mg/dl]). For example, 25% of infants who developed hypoglycaemia spent at least 5 h with interstitial glucose concentrations <2.6 mmol/L [<47 mg/dl], and nearly one-quarter of infants with normal blood glucose concentrations had episodes (≥10 min) of low interstitial glucose concentrations detected only on CGM, some of which were prolonged [3].

At 2 years of age, 404 infants born at ≥35 weeks’ gestation underwent detailed neurodevelopmental assessment [3]. Although hypoglycaemia was not associated with adverse outcome, CGM showed that those with neurodevelopmental impairment had higher glucose concentrations throughout the first 48 h after birth, and a steeper rise in glucose concentrations after hypoglycaemia, particularly if episodes were treated with dextrose rather than feeds alone [3]. These observations suggest that too rapid a rise or higher, less stable blood glucose concentrations during recovery from hypoglycaemia may have adverse effects on the immature brain. This is supported by animal studies showing increased generation of reactive oxygen species and neuronal injury with higher glucose concentrations after hypoglycaemia [48, 49]. Further, when this cohort was reassessed at 4.5 years of age, children who had experienced low glucose concentrations detected by CGM but not by intermittent blood testing had a four-fold increased risk of impaired executive function, whereas the risk in those identified with, and thus treated for, hypoglycaemia was increased only two-fold [50]. This suggests that these clinically undetected changes in glucose concentration may be of physiological significance. More information is needed from CGM about the effect of different treatments and feeding strategies on glycaemic response, glycaemic stability and later outcomes.

### Preterm infants

Since the demonstration that CGM is feasible in very low birth weight babies [51], it has been shown in preterm babies that blood glucose concentrations can fluctuate widely [52] and intermittent blood glucose sampling commonly fails to detect episodes of both hyperglycaemia and hypoglycaemia [14, 53, 54]. Indeed, periods of hypoglycaemia and hyperglycaemia not identified through intermittent glucose blood testing alone have been detected by CGM in up to 50% of preterm babies [2, 5, 55]. Even fully enterally fed preterm babies may have large fluctuations in their glucose concentrations, varying from hypoglycaemia to hyperglycaemia within a day, and for several hours at a time [7, 54]. In one study these fluctuations were related to feed tolerance [8], and in another study episodes of hyperglycaemia were more common in girls and those with fetal growth restriction [54].

The NIRTURE trial of early insulin treatment was the first large trial (*N* = 389) that used CGM without real-time display to detect hyperglycaemia in very low birthweight babies [56]. CGM was well tolerated, even in babies as young as 23 to 24 weeks’ gestation, with minimal interference of nursing cares [51]. Neonatal hyperglycaemia was found to be common, with over half of the babies having a CGM concentration of >10 mmol/L (>180 mg/dl), and was associated with low gestational age and birthweight z-score, and inotrope use [53]. It is important to note that many of the participating centres in this trial used point-of-care glucometers to calibrate the CGM devices, and this will have increased CGM random error [28]. Consequently, there was >10% difference between CGM and point-of-care measures for approximately 25% of recordings, and accuracy relative to true blood glucose (laboratory or blood gas analyser) is unknown [14].

More recently, a randomised controlled trial in very low birthweight infants demonstrated that CGM detected nearly three times as many episodes of hypoglycaemia (<2.8 mmol/L [<50 mg/dl]) during neonatal transition than intermittent blood glucose testing, and the medium duration of each episode was 95 min [5]. However, this study was also limited by use of point-of-care glucometers rather than true glucose measurements for both CGM calibration and diagnosis of hypoglycaemia.

## Clinical use of CGM in neonatal intensive care

While use of CGM in neonatal intensive care is attractive to help improve neonatal glycaemic control and reduced the numbers of blood tests, there is currently little direct evidence of the benefits and risks of this technology in neonates. One small randomised trial (*N* = 43) compared real-time CGM with intermittent blood glucose monitoring in very low birthweight infants and found that CGM reduced the median duration of hypoglycemic episodes <2.8 mmol/L (<50 mg/dl) by 50% (95 vs 44 min) and the number of capillary blood samples by 25% [5]. However, infants in the CGM group also received more intravenous dextrose boluses and total carbohydrate, which could lead to higher or less stable glucose concentrations and increased total fluid intake, factors that were not reported but have been associated with increased morbidity [3, 57]. Thus in absence of clinical outcome data, including neurodevelopmental status, the overall balance of risks and benefits associated with clinical use of CGM in preterm infants remains uncertain.

There are several clinical situations in which neonatal glucose management can be particularly difficult and where optimisation of glycaemic control with CGM may be more likely to improve outcomes. These include infants with prolonged transitional hypoglycaemia, hypoxic ischaemic encephalopathy and preterm infants with hyperglycaemia. There is a paucity of data about use of CGM in these subgroups, and further studies are warranted.

### Prolonged transitional hypoglycaemia

In most term and late preterm infants, hypoglycaemia resolves spontaneously within 1 to 3 days. However, a smaller group of infants experience persistent hypoglycaemia, sometimes called “perinatal stress-induced” or “prolonged transitional” hypoglycaemia, that can last for several weeks before gradually resolving [58]. These infants have dysregulation of pancreatic beta cells with associated hyperinsulinism [59], which can be difficult to manage due to rapidly changing glycaemic status. These infants likely experience significant subclinical hypoglycaemia and are at high risk of neurosensory impairment. A real-time CGM system that warned of impending hypoglycaemia in these infants would potentially allow for a more targeted approach to blood sampling, and may facilitate earlier transition to enteral feeding and reduce exposure to neuroglycopenia.

### Hypoxic ischaemic encephalopathy

Dysglycaemia is common in babies with hypoxic ischemic encephalopathy [60], and both hypo- and hyperglycaemia have been associated with reduced survival and poorer neurological outcome [61]. There is, however, ongoing debate as to whether infants with initial hyperglycaemia have a more [62] or less [63] favourable response to therapeutic hypothermia. Current evidence suggests that the aim of glycaemic management in infants with hypoxic ischaemic encephalopathy should be careful avoidance of hypoglycaemia [64, 65] and maintenance of euglycaemia by regular titration of glucose delivery [65, 66]. This can be difficult to achieve with intermittent testing, unless sampling is frequent. Real-time CGM with trend information may help achieve greater glucose stability, although the effect of whole-body cooling on CGM sensor function is currently not known.

### Hyperglycaemia in preterm infants

In very preterm infants, hyperglycaemia is associated with increased mortality, retinopathy of prematurity, sepsis and long-term neurodevelopmental impairment [67, 68], and animal studies indicate that this association is at least in part causal [69]. Since CGM data has shown that up to half of very low birth weight infants develop hyperglycaemia [2, 53], better detection and treatment of high glucose concentrations may improve outcomes. However, the benefits of treatment with insulin are uncertain as insulin infusions substantially increase the risk of hypoglycaemia, both in very preterm infants [56, 70] and in children in intensive care [71].

The use of combined CGM, insulin pump and computer algorithm (artificial pancreas) has been shown to be effective in type 1 diabetics in reducing the frequency and duration of hypoglycaemia [72]. Computer determined insulin dosing using calculated insulin sensitivity has promise as a management tool in hyperglycaemic preterm infants [73, 74]. However, there are currently no data on the use of CGM to inform insulin management of neonatal hyperglycaemia, although the use of CGM in conjunction with an insulin infusion was reported to reduce the number of episodes of hypoglycaemia in a baby with neonatal diabetes [75]. In paediatric intensive care, use of CGM to guide frequency of blood glucose measurements in a trial of insulin and tight glycaemic control did not prevent severe hypoglycaemia [71]. However, this study did not use calculated insulin sensitivity to guide insulin dose and CGM calibration was based only on point-of-care glucometers without point-to-point calibration. If accuracy of real-time CGM can be improved, it may become possible for very preterm infants with hyperglycaemia to be managed with an artificial pancreas to allow optimal glycaemic control.

## Conclusion

CGM offers considerable potential for optimisation of glycaemia in newborn infants but several issues need to be addressed before this technology can be recommended for real-time monitoring in neonatal intensive care. First, devices should ideally be calibrated to plasma equivalent whole-blood glucose concentrations measured on a blood gas analyser using a point-to-point algorithm. Current CGM devices employ multi-point algorithms that were designed for management of diabetes mellitus in children and adults using home glucometers and at higher glucose concentrations. Real-time calibration methods that are specific to the neonatal intensive care environment are required. Second, the potential for sensor drift, which could result in apparently stable CGM values when blood glucose concentration is falling, requires further investigation, including the extent to which it occurs in neonates and its impact on clinical care. This information will be important in determining how frequently calibration should occur. Third, because CGM has limited point accuracy, clinical metrics should be based on integration of multiple GCM values with a focus on glucose stability, trends and changing metabolic patterns, rather than exceeding specific thresholds. Further research is needed to determine which metrics should be targeted for improving long-term outcomes.

Despite these limitations, retrospective CGM is already an important tool for understanding neonatal glycaemia and the effect of different treatments on glucose metabolism. Current use of CGM should be limited to research studies, and this technology should not be introduced into routine clinical care without evidence of benefit from randomised trials.

## Abbreviations

CGM: Continuous glucose monitor
MARD: Mean absolute relative difference
